# Burdock (*Arctium lappa L*.) leaf flavonoids rich in morin and quercetin 3‐O‐rhamnoside ameliorate lipopolysaccharide‐induced inflammation and oxidative stress in RAW264.7 cells

**DOI:** 10.1002/fsn3.2875

**Published:** 2022-05-12

**Authors:** Jue Cui, Wenyi Zong, Nannan Zhao, Rui Yuan

**Affiliations:** ^1^ 117819 School of Food and Biological Engineering Xuzhou University of Technology Xuzhou China; ^2^ Jiangsu Key Laboratory of Food Resource Development and Quality Safe Xuzhou Institute of Technology Xuzhou China

**Keywords:** anti‐inflammatory, antioxidant, burdock leaf flavonoids, morin, quercetin 3‐O‐rhamnoside

## Abstract

In this study, the anti‐inflammatory and antioxidant activities and mechanism of burdock leaf flavonoids (BLF) on LPS‐stimulated inflammation in RAW264.7 macrophage cells were explored. We have observed that BLF and main effective components morin and quercetin 3‐O‐rhamnoside pretreatment significantly inhibited LPS‐stimulated inflammatory activation of RAW264.7 cells by lowering the levels of NO, PGE2, TNF‐α, and IL‐6 production (*p* < .05). At the same time, BLF not only had potent free radical scavenging ability in vitro (DPPH: 2025.33 ± 84.15 μmol Trolox/g, ABTS: 159.14 ± 5.28 μmol Trolox/g, and ORAC: 248.72 ± 9.74 μmol Trolox/g) but also effectively ameliorated cellular oxidative stress status by restoring the decreased activity of antioxidant enzymes (SOD, CAT, and GSH‐Px) and decreasing the elevated levels of ROS and TBARS in LPS‐stimulated macrophages (*p* < .05). The western blot analysis indicated that BLF and main components morin and quercetin 3‐O‐rhamnoside mainly inhibited LPS‐stimulated inflammation by reducing the iNOS and COX‐2 protein expression, decreasing cellular ROS, and blocking the activation of NF‐κB signaling pathway in macrophages. Our results collectively imply that BLF could be used as a new type of functional factor for the development of antioxidant and anti‐inflammatory foods.

## Graphical abstract

In the present study, we proved that burdock leaf flavonoids treatment could remarkably alleviate LPS‐stimulated oxidative stress and inflammatory response in RAW264.7 cells by restoring the activity of antioxidant enzymes, reducing intracellular ROS content, and blocking NF‐κB signal pathway activation. Q3R and morin are the most important antioxidant and anti‐inflammatory factors of burdock leaf flavonoids. This study offers a theoretical basis for the development of BLF as a natural and effective antioxidant and anti‐inflammatory food additive.

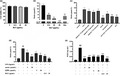

## 1 INTRODUCTION

Inflammation, a natural biological response, could be activated by multiple stimuli such as pathogens, chemical agents, and autoimmune responses (Fujiwara & Kobayashi, 2005). Normally, inflammatory response maintains an equilibrium between anti‐inflammatory and proinflammatory cytokines. However, a growing body of research has found that modern dietary patterns, including high‐fat and high‐sugar diets, could trigger off systemic inflammation (Jamar et al., 2020). The excessive production of proinflammatory mediators has been proved to play a vital role in the progress of chronic inflammatory‐related diseases, such as cancers (Mantovani et al., 2008), metabolic syndromes (Hotamisligil, 2006), atherosclerosis, dermatitis, asthma, and inflammatory bowel diseases (Wan et al., 2019). Therefore, controlling inflammation is an important means to prevent such diseases.

Substantial evidence has shown that macrophages play a central role in high‐sugar diet and high‐fat diet‐induced low‐grade inflammation in the body (Jamar et al., 2020). A number of studies confirmed that macrophages are activated upon consumption of a high‐fat diet. Once activated, macrophage triggers inflammatory response, which is an important cause of insulin resistance in adipocytes (Kawanishi et al., 2010; Lumeng et al., 2007). Studies have found that reduction in macrophage activation could effectively ameliorate diet‐induced insulin resistance (Xiong et al., 2018; Ye et al., 2016). Recent studies have found that many antioxidants can effectively improve the inflammatory state of the body by inhibiting the activation of macrophages (Reshmitha & Nisha, 2021; Zhang et al., 2016). Based on the above research results, we suppose that a novel anti‐inflammatory effector, as a functional factor adding in foods, is in great request for improving diet‐induced chronic inflammation.

Burdock (Arctium lappa L.) is a kind of traditional Chinese medicinal herb and an edible plant. The seeds and roots of burdock are often used to cure inflammation‐related diseases, including arthralgia and throat infections. The anti‐inflammatory activity may lie in that the flavonoids possess high anti‐inflammatory and antioxidant properties, which has highly free radical scavenging ability (Chen et al., 2019; Lin et al., 2021). However, burdock leaves are often discarded as a useless by‐product after burdock harvesting. In our previous study, we found that burdock leaves contain a large number of flavonoids, which have many beneficial biological activities (Cui et al., 2021). Despite burdock leaves possess antioxidant and antibacterial activities, little is known about their anti‐inflammatory activity. In order to develop the utilization value of burdock leaves, it is necessary to study the anti‐inflammatory activity of burdock leaves. Therefore, the objective of the present research is to provide data assistance in the exploitation of a natural food functional factor with anti‐inflammatory and antioxidant activities. The findings of the present study are valuable for using burdock leaf flavonoids as a supplement in health food or functional food.

## 2 MATERIALS AND METHODS

### 2.1 Materials and reagents

DPPH, ABTS, Trolox, fluorescein sodium salt, and lipopolysaccharides (LPS, from Escherichia coli O111:B4) were purchased from Sigma–Aldrich. Morin, quercetin 3‐O‐rhamnoside, kaempferol, apigenin 7‐O‐glucoside, and apigenin 7‐O‐rutinoside were purchased from Shanghai Yuanye Biotechnology Co., Ltd.. TNF‐α and IL‐6 ELISA kits were purchased from Beijing Solarbio Technology Co., Ltd.. NIO detection kit were purchased from Beyotime Biotechnology Co., Ltd.. The antibodies for β‐actin, p65, p‐p65, IκB, p‐IκB, iNOS, COX‐2, and HO‐1 were supplied by Proteintech.

### 2.2 Extraction and purification

Burdock leaf flavonoids extraction, purification, and composition characterization were performed according to our previous research (Cui et al., 2021). In brief, burdock leaf powder was extracted with 60% ethanol in an ultrasonic bath (40 kHz, 200 W) for 40 min at room temperature. The concentrated extract of BLF was purified by a macroporous resin AB‐8 column, and the 70% ethanol eluate of extract was collected, concentrated, lyophilized, and used for compositional analysis and activity evaluation. Flavonoid compositions of BLF were detected using an UPLC–QTOF system. The major components were characterized by comparing the current mass data and retention time, and the peak area was used to calculate the relative content of each compound. Seven main flavonoids in BLF were identified, including apigenin 7‐O‐apiosyl‐glucoside (10.23%), apigenin 7‐O‐glucoside (8.27%), apigenin 7‐O‐rutinoside (14.89%), morin (16.77%), kaempferol (9.13%), kaempferol 3,7‐O‐diglucoside (5.50%), and quercetin 3‐O‐rhamnoside (8.44%), and these flavonoids account for 73.23% of the BLF.

### 2.3 Total flavonoid content of BLF

Total flavonoid content of crude BLF and purified BLF were determined according to one previous method with few modifications (Bajalan et al., 2016). 2.7 ml of sample solution and 0.15 ml of sodium nitrite solution (5%, w/v) were blended, and the reaction mixture was cultured at 37°C for 6 min in the dark. After incubation, the reaction system was incubated for 6 min after adding 0.15 ml of aluminum nitrate solution (10%, w/v). Subsequently, 2 ml of sodium hydroxide (1 mol/L) was added into the mixed solution and incubated for 10 min. Finally, the absorbance of the reaction solution was detected at 510 nm, and the flavonoids content of samples was showed as mg of rutin equivalent (RE) per gram of the freeze‐dried BLF sample using a rutin calibration curve.

### 2.4 DPPH and ABTS free radicals scavenging assay

The scavenging activities of BLF on DPPH and ABTS free radical were examined according to the previous method (Bajalan et al., 2016; Tyagi et al., 2021). Trolox was used for positive control, and the results of free radical scavenging assay were indicated as Trolox equivalent relative to sample (μmol Trolox/g).

### 2.5 Oxygen radical absorbance capacity assay

Oxygen radical absorbance capacity (ORAC) assay was examined based on one previous method with some modifications (Rodríguez‐Bonilla et al., 2017). In brief, 20 μl of sample solutions for different concentrations, 20 μl of sodium fluorescein solution (63 nmol/L), and 20 μl of PBS buffer (0.075 mol/L, pH 7.4) were mixed and added to a 96‐well microplate. The mixed solutions were incubated at 37°C. After incubation for 15 min, 140 μl of AAPH solution (20 mmol/L) was added to the reaction system. The fluorescence values of the reaction system were detected every 2 min until all the fluorescence values were extinction. ORAC value of samples was showed as Trolox equivalent relative to sample (μmol Trolox/g) using Trolox as a positive control.

### 2.6 Cell culture and cytotoxicity assay

Murine macrophage cell RAW264.7 was purchased from the Cell Bank of the Chinese Academy of Sciences (Shanghai, China) and cultured in DMEM added 10% FBS and streptomycin/penicillin (100 U/ml) at 37°C in a 5% CO_2_ incubator. The RAW264.7 cells (1 × 10^5^ cells/well) were incubated with samples at different concentrations at 37°C. After culturing for 20 h, the CCK‐8 solution (Beyotime) was added to the culture solution, and the RAW264.7 cells were further cultured for another 4 h. Finally, the absorbance of the cell culture medium was read at 450 nm.

### 2.7 Nitric oxide determination

The nitric oxide (NO) concentrations of cell supernatant were detected using the Griess method as described previously (Qu et al., 2018). RAW264.7 cells were cultivated in a cell cultured plate (1 × 10^5^ cells/well) for 24 h. After culturing for 12 h, the cells were pretreated with BLF at different concentrations for 2 h, and the cells were activated with LPS (1 μg/ml) for 24 h at 37°C, respectively. Dexamethasone (1 μmol/L) was used as a positive control. After incubation for 24 h, to determine the NO levels, 100 μl of cell supernatant and 100 μl of Griess reagent were mixed, and the absorbance of the mixture was detected at 540 nm. The nitrite concentration was calculated according to the regression equation of the standard curve.

### 2.8 Determination of oxidative stress status

The RAW264.7 cells were cultured in a six‐well plate and pretreated with different concentrations of BLF (12.5 and 25 μg/ml), morin (5 and 20 μmol/L), and quercetin 3‐O‐rhamnoside (5 and 20 μmol/L) for 2 h, respectively. The cells were activated with LPS (1 μg/ml) for 24 h. After culturing for 24 h, the activities of superoxide dismutase (SOD), catalase (CAT), and glutathione peroxidase (GSH‐Px) were measured using assay kits, respectively, following the manufacturer's instructions (Nanjing Jiancheng). The enzyme activities were indicated as unit/mg protein. To assess the level of lipid oxidation in the cells, the thiobarbituric acid reacting substance (TBARS) of the cells were measured using assay kits, following the manufacturer's instructions (Elabscience).

### 2.9 Intracellular ROS levels assay

The levels of intracellular ROS for RAW264.7 cells were measured using DCFH‐DA staining. After pretreatment, the cells were incubated with DCFH‐DA for 20 min and washed twice with PBS. The fluorescence value of the cells was measured using a fluorescence spectrophotometer (Synergy HT, Bio‐Tek).

### 2.10 Inflammatory cytokines and PGE2 production determination

The levels of IL‐6, TNF‐α, and PGE2 of supernatant for the cells were measured using ELISA kits (Solarbio), respectively, according to the manufacturer's instructions.

### 2.11 Western blotting analysis

After treatment, the cells were lysed with RIPA solution mixed with phosphatase and protease inhibitors. After centrifugation, the supernatant was quantified using the Bradford method. The protein of the cells was separated with 10% SDS‐PAGE and transferred to the NC membrane. The NC membrane was blocked using skim milk for 2h and incubated with a primary antibody (Proteintech), including β‐actin, COX‐2, iNOS, HO‐1, p‐IκB, IκB, NF‐κB p65, and NF‐κB p‐p65 at 4°C overnight. After incubation, the membrane was washed with TBST and incubated with secondary antibody (Proteintech) for 2 h. The protein bands were inspected using a ECL kit (Millipore), and the bands image was captured with a ChemiDoc imaging system and quantified by densitometry using ImageJ software (National Institutes of Health) with β‐actin as the loading control.

### 2.12 Statistical analysis

All experiments in this study were repeated at least three times and the results were expressed as means ±standard deviations (*SD*). One‐way analysis of variance (ANOVA) to evaluate statistical significance of differences and Tukey post‐hoc tests were performed with GraphPad Prism V.8.0 (GraphPad Software Inc.).

## 3 RESULTS AND DISCUSSION

### 3.1 Flavonoid content and in vitro antioxidant activities of BLF

To evaluate the antioxidant activity of BLF, firstly, the flavonoids content and the scavenging capacities of crude BLF and purified BLF on DPPH and ABTS free radical in vitro were investigated. The flavonoids content of crude BLF and purified BLF were 73.28 ± 3.15 and 239.94 ± 10.52 mg RE/g, respectively. The scavenging capacities of DPPH and ABTS radical for crude BLF and purified BLF were expressed as Trolox equivalents. The result of the DPPH assay showed that purified BLF (2025.33 ± 84.15 μmol Trolox/g) showed higher scavenging activity than crude BLF (577.10 ± 23.62 μmol Trolox/g). The result of the ABTS assay showed that purified BLF (159.14 ± 5.28 μmol Trolox/g) exhibited higher activities than crude BLF (49.31 ± 2.36 μmol Trolox/g). In the same way, ORAC values of crude BLF and purified BLF were evaluated to be 73.26 ± 3.27 and 248.72 ± 9.74 μmol Trolox/g, respectively. The results of free radical scavenging activity and flavonoids content indicated that purified BLF showed stronger antioxidant activity and flavonoids content than crude BLF, implying that the antioxidant activities in vitro increased as the concentrations of flavonoids content increased (Table 1).

**TABLE 1:** Total flavonoid content and antioxidant activity in vitro of BLF

	Total flavonoids content (mg RE/g)	DPPH (μmol Trolox/g)	ABTS (μmol Trolox/g)	ORAC (μmol Trolox/g)
Crude BLF	72.38 ± 3.15	577.10 ± 23.62	49.31 ± 2.36	73.26 ± 3.27
Purified BLF	239.94 ± 10.52	2025.33 ± 84.15	159.14 ± 5.28	248.72 ± 9.74

### 3.2 BLF reduced PGE2 and NO production of LPS‐activated RAW264.7 cells

**FIGURE 1:**
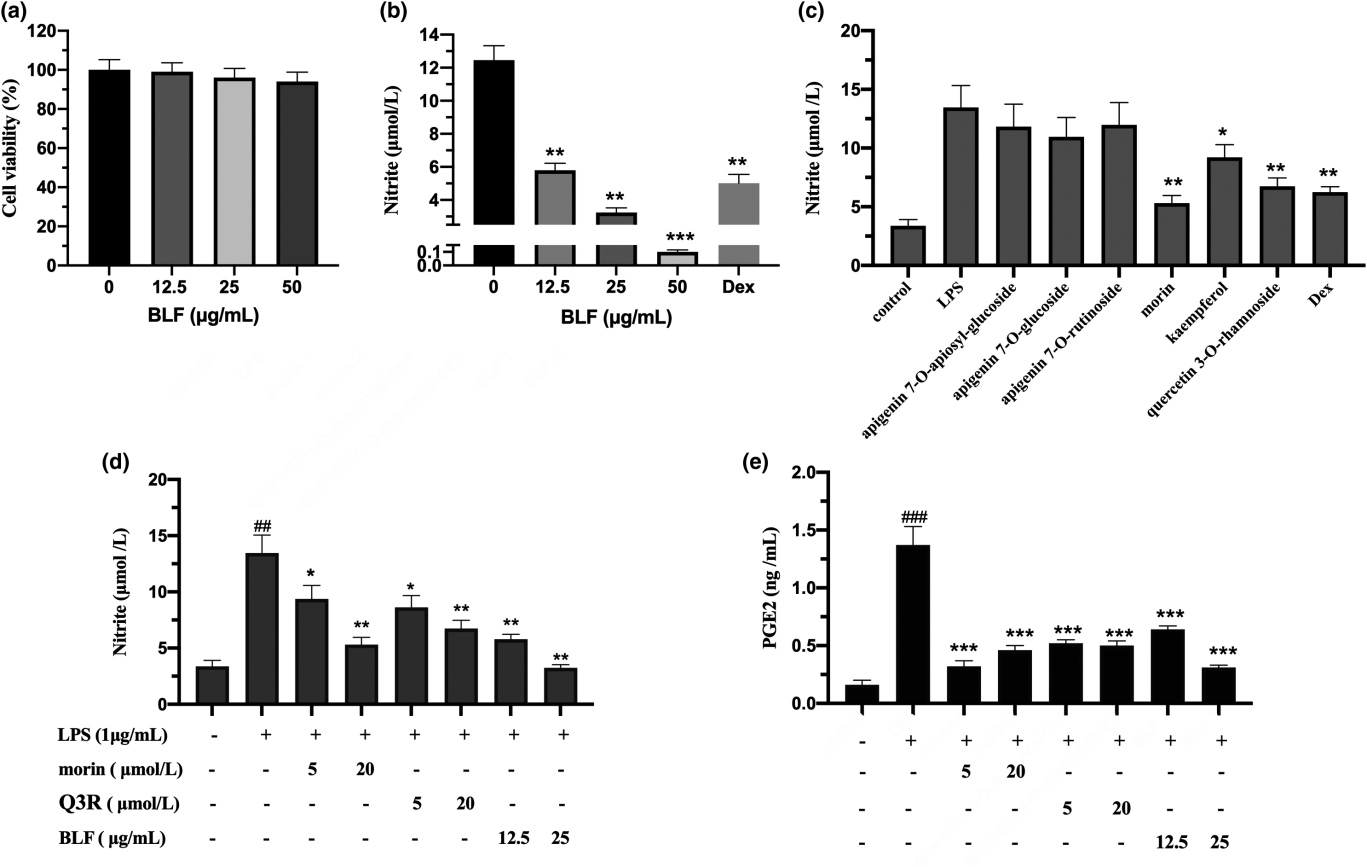
BLF and its main components reduced PGE2 and NO production of LPS‐activated RAW264.7 cells. (a) Cytotoxic effect of BLF on RAW264.7 cells. (b) Inhibitory effect of BLF on NO production of LPS‐activated RAW264.7 cells. **p < .01, ***p < .001 in comparison with LPS‐treated cells. (c) Inhibitory effect of main components for BLF on NO production of LPS‐activated RAW264.7 cells. *p < .05, **p < .01 in comparison with LPS‐treated cells. (d) Inhibitory effect of morin, quercetin 3‐O‐rhamnoside (Q3R), and BLF on NO production of LPS‐activated RAW264.7 cells. ##*p* < .01 in comparison with untreated cells. (e) Inhibitory effect of morin, Q3R, and BLF on PGE2 production of LPS‐activated RAW264.7 cells. ###*p* < .001 in comparison with untreated cells

Nitric oxide is a critical signaling molecule that is generated by nitric oxide synthase, and it has been confirmed to play a vital role in the development of inflammatory‐related diseases (Xue et al., 2020). Therefore, in the present study, anti‐inflammatory activities of BLF were estimated by detecting NO production of LPS‐stimulated RAW264.7 cells. Firstly, the cytotoxic effect of BLF was evaluated at different concentrations on LPS‐activated RAW264.7 cells. Results of cell viability following treatment with various concentrations for 24 h were showed in Figure 1. As shown in Figure 1a, no manifest decrease in cell viability was observed after treatment with BLF at the tested concentration, indicating that BLF had no cytotoxic effects on RAW264.7 cells within the tested concentration. To evaluate the inhibiting activity of BLF on NO secretion levels, the NO concentrations in the cell supernatant were measured by Griess assays (Figure 1b). BLF can dose dependently suppress the NO production of LPS‐stimulated macrophages (*p* < .01). To elucidate the effective ingredients of BLF, the compound composition and the inhibiting activity of the main ingredients of BLF on NO secretion levels of LPS‐stimulated RAW264.7 cells were estimated. The results of UPLC‐QTOF showed that the main components in BLF included morin, several apigenin glycosides, quercetin glycoside, kaempferol, and its glycoside. The inhibiting activity of main ingredients of BLF on NO secretion levels showed that the NO production was significantly decreased by quercetin 3‐O‐rhamnoside and morin at 20 µmol/L (*p* < .01), while the inhibitory effects of apigenin glucosides were not significant. To further substantiate the anti‐inflammatory effects of quercetin 3‐O‐rhamnoside (Q3R) and morin, the NO and PGE2 concentrations in the cell culture medium were measured. As shown in Figure 1d,e, pretreatment of cells with morin, Q3R, and BLF at tested concentrations dose dependently inhibited NO (*p* < .05) and PGE2 (*p* < .001) production of LPS‐activated RAW264.7 cells, suggesting that Q3R and morin could be the main anti‐inflammatory ingredients in BLF.

### 3.3 BLF inhibited ROS and TBARS production of LPS‐activated RAW264.7 cells

**FIGURE 2:**
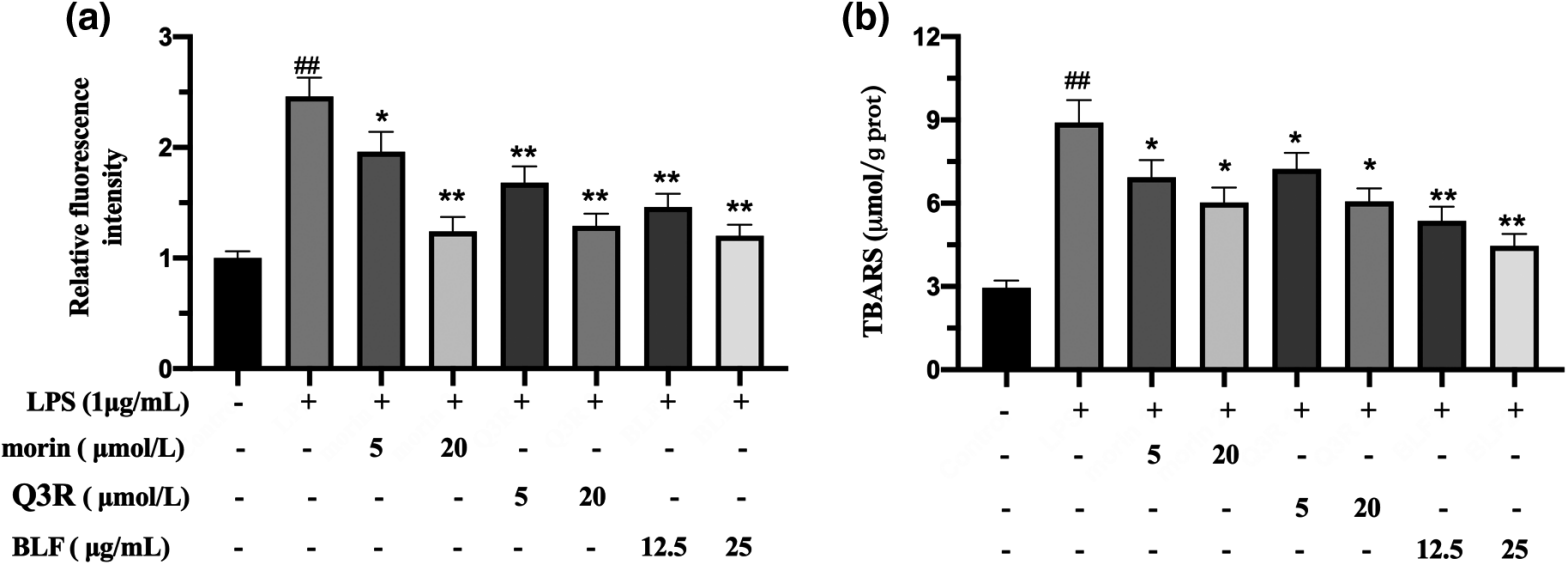
BLF, morin, quercetin 3‐O‐rhamnoside (Q3R) inhibited ROS and TBARS production of LPS‐Induced RAW264.7 Cells. (a) ROS content of the RAW264.7 Cells (b) TBARS content of the RAW264.7 Cells. ##*p* < .01 in comparison with untreated cells. *p < .05, **p < .01 in comparison with LPS‐treated cells

Several studies have confirmed that reactive oxygen species (ROS) are closely related to the pathogenesis of chronic inflammatory diseases (El‐Kenawi & Ruffell, 2017). As shown in Figure 2a, the results of ROS production indicated that pretreatment of RAW264.7 cells with BLF, morin, and Q3R at tested concentrations significantly inhibited LPS‐stimulated ROS excessive production (*p* < .05). TBARS, the product of lipid peroxidation for unsaturated fatty acids after the free radical attack, is commonly used as a biomarker of oxidative stress (Tsikas, 2017). In this study, the extent of lipid peroxidation in cells was analyzed by TBARS, and the results of TBARS showed that supplementation of morin, Q3R, or BLF effectively suppressed LPS‐stimulated TBARS concentration elevation (Figure 2b). The TBARS contents of morin (5 µmol/L), Q3R (5 µmol/L) and BLF (12.5 µg/ml) groups effectively decreased to 6.92, 7.23, and 5.36 µmol/g prot, respectively. Twenty µmol/L of morin and Q3R, and 25 µg/ml of BLF pretreatment groups significantly decreased the TBARS generation down to 6.35, 6.41, and 4.47 µmol/g prot, respectively (*p* < .05), suggesting that BLF could effectively eliminate LPS‐stimulated ROS production and lipid peroxidation in cells.

### 3.4 BLF improved activity of antioxidant enzymes in LPS‐activated RAW264.7 cells

**FIGURE 3:**
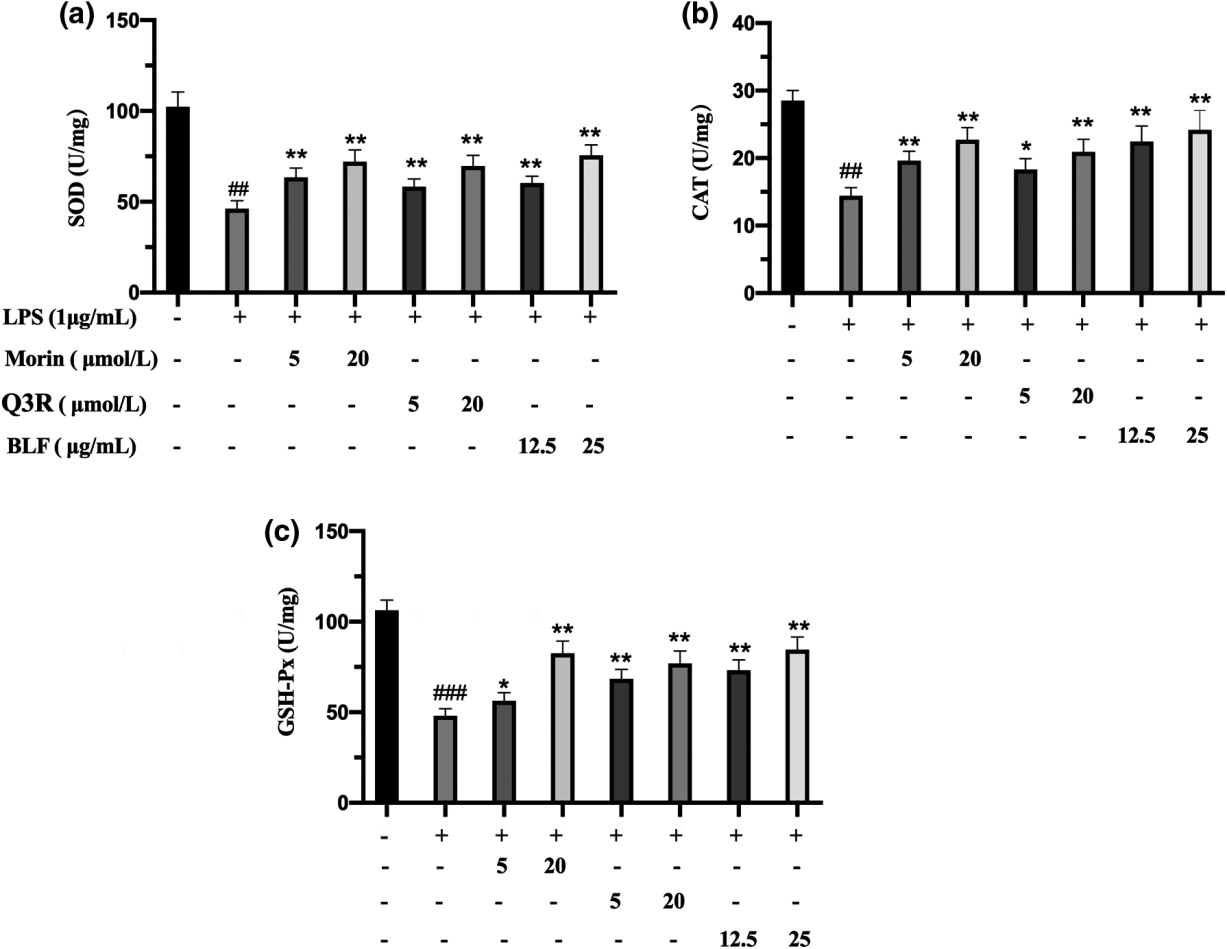
BLF, morin, and Q3R improved the activity of antioxidant enzymes in LPS‐activated RAW264.7 cells. (a) SOD activity of the RAW264.7 Cells (b) CAT activity of the RAW264.7 Cells (c) GSH‐Px activity of the RAW264.7 Cells

SOD, CAT, and GSH‐Px are important enzymes for ROS scavenging in cells, and these enzymes could eliminate LPS‐stimulated ROS production and lessen cell damage induced by oxidative stress (Liu et al., 2011). In this study, LPS induced a decrease for vitalities of SOD, CAT, and GSH‐Px. BLF, morin, and Q3R pretreatment restored activities of all the tested enzymes. As shown in Figure 3a–c, the vitalities of SOD, CAT, and GSH‐Px in control groups were detected to be 102.5, 28.5, and 106.3 U/mg, while the enzymatic activities of the LPS groups decreased to 46.2, 14.4, and 48.1 U/mg, respectively. Pretreatment with BLF, morin, and Q3R significantly restored the vitalities of all the tested enzymes in LPS‐activated RAW 246.7 cells (*p* < .01), respectively. The above results implied that scavenging of excessive ROS and restoring the activity of antioxidant enzymes may be the important pathways for BLF to inhibit the inflammatory activation induced by LPS in RAW246.7 cells.

### 3.5 BLF decreased the level of inflammatory cytokines in LPS activated RAW264.7 cells

**FIGURE 4:**
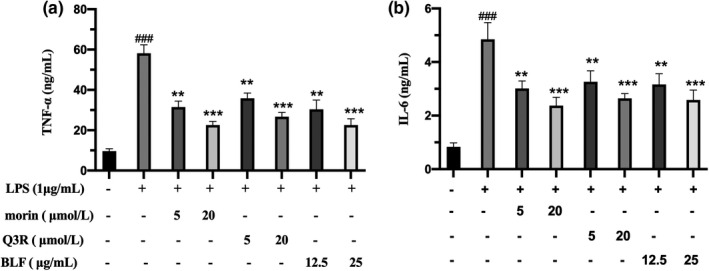
BLF, morin, and Q3R inhibited the production of TNF‐α and IL‐6 in LPS‐activated RAW264.7 cells. (a) The TNF‐α concentration of supernatant for the RAW264.7 Cells. (b) IL‐6 concentration of supernatant for the RAW264.7 Cells

Previous researches have confirmed that LPS stimulation can promote the generation of IL‐6, TNF‐α, and other inflammatory cytokines. These inflammatory cytokines play a crucial role in the initiation and development of inflammation (Lee et al., 2017; Wellen & Hotamisligil, 2005; Xu et al., 2020). TNF‐α, a proinflammatory cytokine secreted by activated macrophages, can activate NF‐κB signaling pathway to regulate the expression of a series of inflammatory factors (Cheon, 2017). IL‐6 is produced by activated macrophages, and dysregulated continual production of IL‐6 plays a vital role in chronic inflammatory response and autoimmunity (Mohamad et al., 2018). To assess the impact of BLF on the generation of inflammatory cytokines in RAW264.7 cells, the concentrations of TNF‐α and IL‐6 were detected by ELISA. As shown in Figure 4, BLF, morin, and Q3R markedly inhibited RAW264.7 macrophages from releasing TNF‐α and IL‐6 within the tested concentrations (*p* < .01). Compared with the LPS treatment group, when macrophages were treated with 20 µmol/L of morin, the concentrations of TNF‐α and IL‐6 were significantly decreased to 22.59 and 2.37 ng/ml, respectively. Similarly, Q3R has shown effective inhibition of inflammatory factor secretion. As compared with the LPS group, when macrophages were treated with 20 µmol/L of Q3R, the concentrations of TNF‐α and IL‐6 were decreased significantly, being 26.70 and 2.64 ng/ml, respectively. Similar results were reported by those who found that morin could significantly eliminate monosodium urate crystal‐stimulated inflammatory response in RAW264.7 cells (Dhanasekar et al., 2015). Likewise, several studies have substantiated the anti‐inflammatory activities of morin (Case lli et al., 2016; Guerra et al., 2006; Li et al., 2016, 2020; Tianzhu et al., 2014; Wang et al., 2016). These studies demonstrated that morin is an effective anti‐inflammatory agent that inhibits most effectors involved in inflammation by inhibiting activated macrophages, both in vitro and in vivo. Based on the above results, it can be suggested that BLF could significantly suppress LPS‐induced generation of TNF‐α and IL‐6 for activated macrophages, and morin and Q3R may be the key active components of BLF.

### 3.6 BLF inhibited activation of the NF‐κB signaling pathway induced by LPS

**FIGURE 5:**
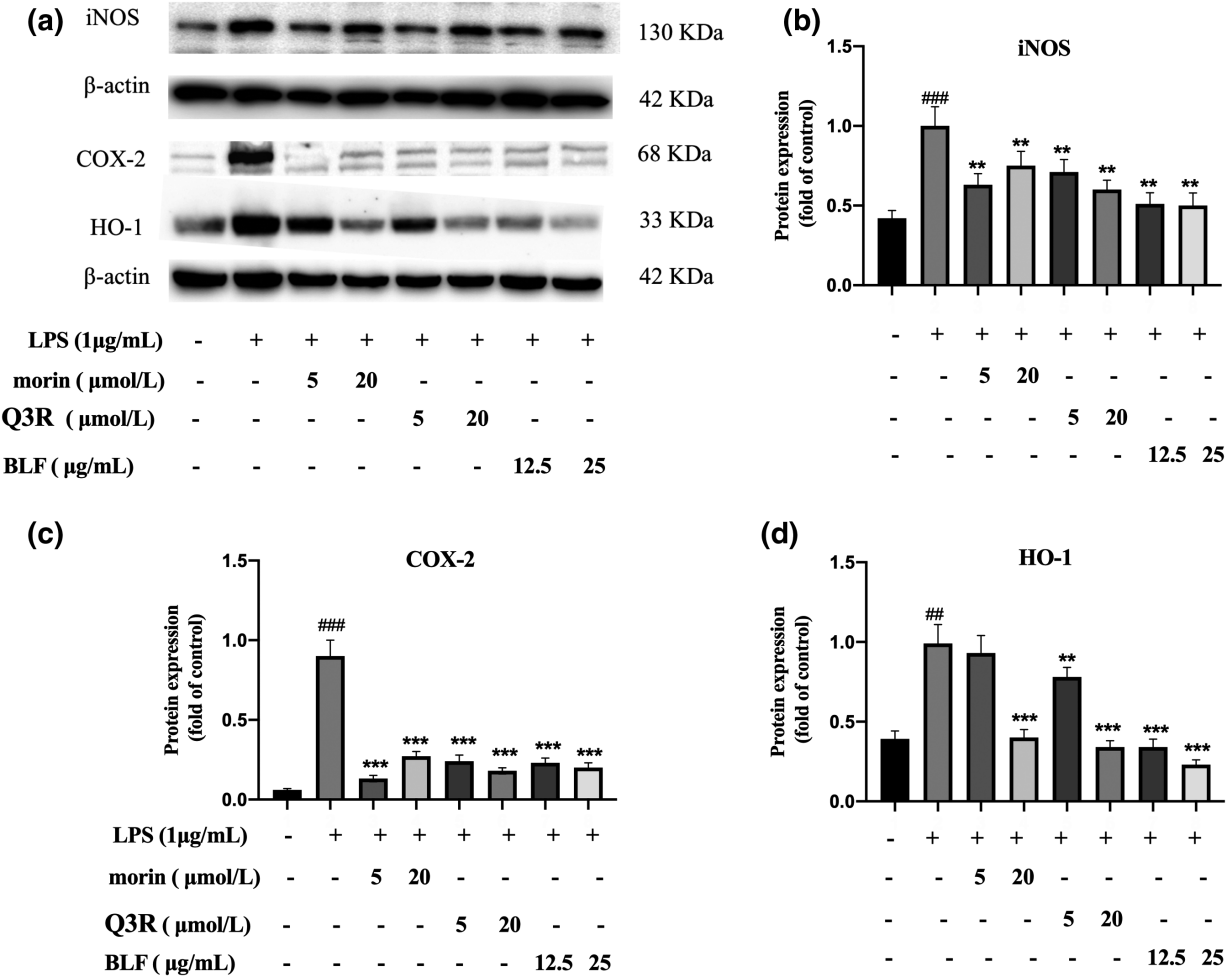
Protein expression levels of iNOS, COX‐2, and HO‐1 of the RAW264.7 cells. (a) The protein bands photograph of the RAW264.7 cells. (b) Relative protein expression of iNOS for the RAW264.7 cells. (c) Relative protein expression of COX‐2 for the RAW264.7 cells. (d) Relative protein expression of HO‐1 for the RAW264.7 cells. ##*p* < .01, ###*p* < .001 in comparison with untreated cells. **p < .01, ***p < .001 in comparison with LPS‐treated cells

Based on the results of the BLF‐induced reduction in PGE2 and NO generation, we detected the protein expression levels of COX‐2 and iNOS. COX‐2 and iNOS are the important downstream factors of the NF‐κB signaling pathway and the crucial enzymes of NO and PGE2 generation (Liu et al., 2017). As shown in Figure 5a,b, pretreatment with BLF significantly reduced the LPS‐induced elevation of COX‐2 (*p* < .001) and iNOS protein (*p* < .01), indicating that BLF‐inhibited LPS‐stimulated generation of PGE2 and NO in RAW264.7 cells by downregulating protein expression levels of COX‐2 and iNOS at the translation levels. Similarly, the two key active components in BLF, including Q3R and morin, also decreased protein levels of COX‐2 and iNOS protein, indicating that morin and Q3R may be the key active components for the anti‐inflammatory activity of BLF.

Nuclear factor (erythroid‐derived 2)‐like 2 (Nrf2) is a pivotal eukaryotic redox‐active effector, and HO‐1 is an important downstream effector protein of Nrf2, which is expressed to protect cells against ROS attack (Loboda et al., 2016). Therefore, we assessed oxidative stress status by detecting HO‐1 protein levels in RAW 264.7 cells. As shown in Figure 5c, LPS stimulation significantly increased the expression of HO‐1 protein, indicating that LPS stimulation can increase the level of intracellular ROS, resulting in oxidative stress. Pretreatment with BLF significantly reduced the LPS‐induced elevation of HO‐1 protein (*p* < .001), suggesting that BLF does not play a protective role by stimulating Nrf2‐HO‐1 signaling pathway, but alleviates LPS‐induced oxidative stress in RAW264.7 cells by eliminating redundant ROS.

**FIGURE 6:**
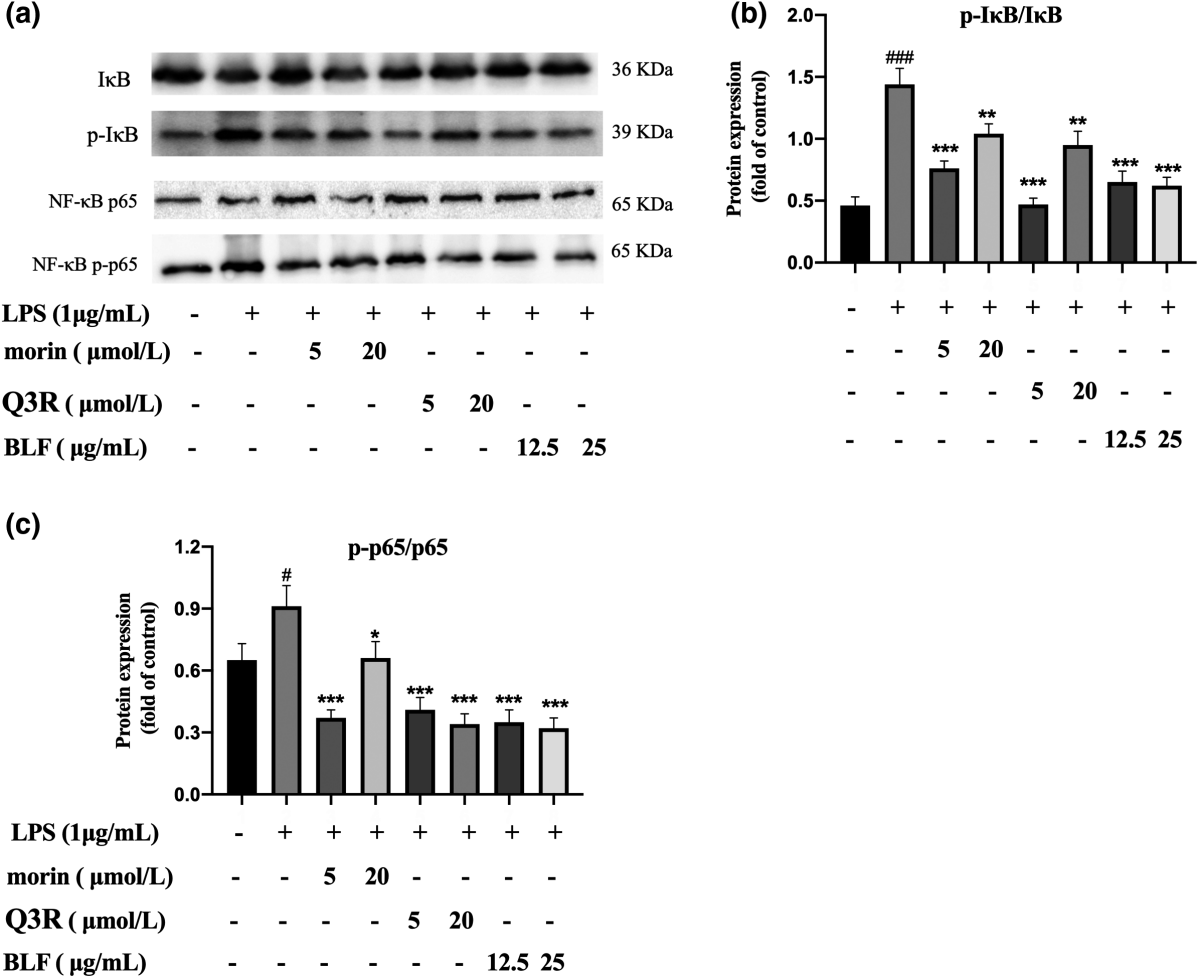
Protein expression levels of IκBα, p‐IκBα, p65, and p‐p65. (a) The protein bands photograph of the RAW264.7 cells. (b) Relative protein expression of p‐IκBα/IκBα for the RAW264.7 cells. (c) Relative protein expression of p‐p65/p65 for the RAW264.7 cells. #*p* < .05, ###*p* < .001 in comparison with untreated cells. *p < .05, **p < .01, ***p < .001 in comparison with LPS‐treated cells

NF‐κB is a pivotal signaling pathway controlling the synthesis and release of proinflammatory cytokines and mediators, such as iNOS, PGE2, TNF‐α, and IL‐6, in the inflammatory response (Liu et al., 2017). Under normal conditions, in the absence of inflammatory stimulation, NF‐κB is inactivated by entanglement in the cytoplasm to form a complex with its inhibitor IκB. When stimulated by inflammation, IκBα is phosphorylated and degraded, resulting in the release of NF‐κB p65. NF‐κB p65 is an important subunit in charge of promoter binding and transcriptional regulation of multiple inflammatory genes. When IκBα is phosphorylated and degraded, p65 is phosphorylated and transferred into the cell nucleus to initiate transcription of multiple proinflammatory mediators and cytokines. Therefore, phosphorylation of IκB‐α and p65 is important for the expression of cytokines and inflammatory mediators. To explore the mechanism of BLF inhibiting macrophage activation, protein expression levels of IκBα, p‐IκBα, p65, and p‐p65 were determined. As shown in Figure 6, LPS stimulation effectively elevated protein levels of p‐IκBα/IκBα (*p* < .001) and p‐p65/p65 (*p* < .05) of RAW264.7 cells, indicating that LPS activation effectively promotes activation of NF‐κB signaling pathway. Simultaneously, BLF pretreatment remarkably reduced protein levels of p‐IκBα/IκBα and p‐p65/p65 (*p* < .001), implying that BLF could inhibit the gene expressions and release inflammatory cytokines and proinflammatory mediators by inhibiting LPS‐activated NF‐κB signal pathway activation. Similar results were reported by those who found that morin inhibits the activity of the transcription factor NF‐kB by stabilizing IkBα, thus reducing the expression of inducible form of iNOS, as well as of the COX‐2, IL‐6, and TNF genes (Manna et al., 2007).

The above results suggested that BLF could effectively alleviate the LPS‐promoted inflammatory reaction in RAW264.7 cells by inhibiting NF‐κB signaling pathway activation. The main anti‐inflammatory components of BLF are Q3R and morin.

## 4 CONCLUSIONS

In the present study, we proved that BLF treatment could remarkably alleviate LPS‐stimulated oxidative stress and inflammatory response in RAW264.7 cells by restoring the activity of antioxidant enzymes, reducing intracellular ROS content, and blocking NF‐κB signal pathway activation. Q3R and morin are the most important antioxidant and anti‐inflammatory factors of BLF. This study offers a theoretical basis for the development of BLF as a natural and effective antioxidant and anti‐inflammatory food additive.

## ETHICAL APPROVAL

Ethics approval was not required for this research.

## Data Availability

The data that support the findings of this study are available on request from the corresponding author. The data are not publicly available due to privacy or ethical restrictions.
